# A Deep Learning-Based Model for Classification of Different Subtypes of Subcortical Vascular Cognitive Impairment With FLAIR

**DOI:** 10.3389/fnins.2020.00557

**Published:** 2020-06-18

**Authors:** Qi Chen, Yao Wang, Yage Qiu, Xiaowei Wu, Yan Zhou, Guangtao Zhai

**Affiliations:** ^1^Institute of Image Communication and Network Engineering, Shanghai Jiao Tong University, Shanghai, China; ^2^Department of Radiology, Renji Hospital, School of Medicine, Shanghai Jiao Tong University, Shanghai, China

**Keywords:** subcortical ischemic vascular disease, convolutional neural network, deep learning, magnetic resonance imaging, cognitive impairment

## Abstract

Deep learning methods have shown their great capability of extracting high-level features from image and have been used for effective medical imaging classification recently. However, training samples of medical images are restricted by the amount of patients as well as medical ethics issues, making it hard to train the neural networks. In this paper, we propose a novel end-to-end three-dimensional (3D) attention-based residual neural network (ResNet) architecture to classify different subtypes of subcortical vascular cognitive impairment (SVCI) with single-shot T2-weighted fluid-attenuated inversion recovery (FLAIR) sequence. Our aim is to develop a convolutional neural network to provide a convenient and effective way to assist doctors in the diagnosis and early treatment of the different subtypes of SVCI. The experiment data in this paper are collected from 242 patients from the Neurology Department of Renji Hospital, including 78 amnestic mild cognitive impairment (a-MCI), 70 nonamnestic MCI (na-MCI), and 94 no cognitive impairment (NCI). The accuracy of our proposed model has reached 98.6% on a training set and 97.3% on a validation set. The test accuracy on an untrained testing set reaches 93.8% with robustness. Our proposed method can provide a convenient and effective way to assist doctors in the diagnosis and early treatment.

## Introduction

Vascular cognitive impairment (VCI) is a broad term that includes a group of cognitive disorders with various degrees of severity, from mild to severe attributable to pathological damage of the cerebral vascular system (Barbay et al., 2017). Vascular dementia developed from VCI is the second most common cause of dementia after Alzheimer’s disease (AD) (Barbay et al., 2017). Recently, VCI, especially its most common form subcortical VCI (SVCI), has been getting increased attention, for there is increasing evidence that impaired vascular structure and function are also important in the development of AD (Lucy et al., 2017). SVCI is defined as a clinical continuum of cognitive impairments due to cerebral small vessel disease (Olivia et al., 2018). Lacunar infarct and white matter hyperintensities (WMHs) (also termed white matter lesions or leukoaraiosis), which are located subcortically or deeply), are the main type of lesions. Prominent perivascular spaces, cerebral microbleeds, and atrophy are the other common signs shown in conventional MRI sequences that are associated with SVCI (Jee and Lee, 2014). Nowadays, with the development of neuroimaging studies, we have gradually found that conventional MRI characteristics cannot fully explain the variable clinical manifestations of SVCI. For example, although voxel-based morphometry and lesion-symptom mapping studies have shown extensive brain damages in SVCI patients, the relationship between these damages and clinical cognitive impairments is still controversial among different studies (Marco et al., 2011; Biesbroek et al., 2013, 2017). The International Society for Vascular Behavior and Cognitive Disorders suggested that a strategic infarct or hemorrhage, multiple lacunes, one large infarct or hemorrhage, and extensive and confluent WMH of vascular origin may be helpful in the diagnosis of SVCI (Perminder et al., 2014). However, as there is little validation of these thresholds, the exact clinical relevance patterns for individual patients remain to be discussed. So by now, the diagnosis of SVCI still relies on scrupulous clinical assessment such as detailed medical history enquiry, physical and neurobiological exams, and neuropsychological evaluation, which are costly, are time-consuming, are subjectively dependent, and may even be traumatic. More effective methods to classify and evaluate the cognitive impairments of SVCI are needed.

Noticeably, a small number of studies have made an effort to resolve the dilemma by traditional machine learning (ML) based on neuroimaging data. Using hierarchical fully convolutional network (H-FCN), Lian et al. (2020) automatically identified discriminative atrophy local patches and regions in brain structural MRI (sMRI) and achieve state-of-the-art AD versus normal control (NC) and progressive mild cognitive impairment (pMCI) versus stable MCI (sMCI) classification performance. By combining diffusion tensor imaging (DTI) and brain morphometry parameters, Stefano et al. (2015) successfully discriminated healthy controls from patients with vascular dementia and vascular MCI (VaMCI) by ML techniques. Stefano et al. (2016) adopted a support vector machine (SVM)-based ML strategy for discrimination SVCI patients with different cognitive performances on the basis of predefined feature vectors extracted from DTI data. The sensitivity, specificity, and accuracy of the classification model were 72.7–89.5%, 71.4–83.3%, and 77.5–80.0%, respectively. Finally, except for not being sensitive enough, extracting those features on the basis of such large data volume of neuroimaging needs human experts, which are often costly, time-consuming, and burdensome. Deep learning (DL) is a rapidly developing ML algorithm for directly extracting high-throughput features from the images without the engagement of human experts. In particular, quite a lot of studies focus on the application of DL-based diagnosis assistance system. Duan et al. (2019) have researched the visual attention analysis of children with autism spectrum disorder (ASD). Liu et al. (2019) focused on the AD diagnosis and used deep multi-task multi-channel learning to achieve state-of-the-art classification results. Yang et al. (2020b) fused deep spatial and temporal features from adaptive dynamic functional connectivity (dFC) and achieved great classification accuracy of 87.7%, which is 5.5% higher than that of the state-of-the-art methods. Liu et al. (2018) proposed a deep multi-instance convolutional neural network (CNN) to automatically learn both local and global representations for MR images and achieve superior performance over state-of-the-art approaches. In particular, in MCI classification problems, Yang et al. (2014, 2020a) have proposed effective sparse functional connectivity networks and sparse multivariate autoregressive modeling methods for MCI classification. In our previous study (Yao et al., 2019), we trained a CNN to classify different cognitive performances in patients with subcortical ischemic vascular disease (SIVD) on the basis of T2-weighted fluid-attenuated inversion recovery (FLAIR) data. For the three-dimensional (3D)-based model, the accuracy of a training set and a testing set reached 99.7 and 96.9%, respectively. This previous study suggests us that DL, especially 3D-CNN, is a powerful and convenient method for classification of SVCI by single-shot T2-weighted FLAIR sequence. By focusing on the sparse regression of blood oxygenation level dependent (BOLD) MRI and arterial spin labeling (ASL) MRI as well as the brain connectivity network inferred from the MR image, Li et al. (2019) and Yang et al. (2019) proposed novel state-of-the-art methods on MCI classification.

With the successful use of 3D-CNN in classifying different stages of cognitive impairment in SVCI, we decided to further our study and refine the model for classifying different subtypes of VaMCI on the basis of the single-shot FLAIR sequence. VaMCI is an intermediate and reversible state between normal cognitive status and vascular dementia. The definition of MCI according to criteria proposed by a multidisciplinary and international experts group includes four clinical subtypes: amnestic MCI (a-MCI; single or multiple domain) and nonamnestic MCI (na-MCI; single or multiple domain) (Winblad et al., 2004). Different VaMCI subtypes might subtend different etiologies: a-MCI (single or multiple domains) was considered to have a degenerative etiology, and multidomain MCI (either amnestic or not) was considered to have a vascular etiology (Emilia et al., 2016). The subtypes of VaMCI are important for clinical care and targeted treatment and might be associated with prognosis. David et al. (2015) found that dementia risks were higher for a MCI than for na-MCI, and for multidomain compared with single-domain MCI.Liesbeth et al. (2017) found that the relevance of reversion for progression risk depends on the MCI subtype. The risk of dementia in participants with MCI who did not revert, especially in amnestic subtype, was higher than in reverters. Neuroimaging studies showed some signs in differentiating a-MCI and na-MCI. Yukako et al. (2019) found that medial temporal lobe atrophy and lower educational history are quick indicators of amnestic cognitive impairment after stroke. Another study showed that medial temporal lobe atrophy was more frequent in multidomain compared with single domain (Emilia et al., 2016). Hosseini et al. (2017) compared different subtypes of VCI on the basis of DTI and FLAIR data. Results showed that higher medial temporal lobe atrophy and left hippocampal mean diffusivity contributed to amnestic VCI and that higher ischemic burden contributed to nonamnestic VCI.

Considering the importance of VaMCI subtypes for clinical decision, and the possibility for image classification suggested by limited neuroimaging studies, we constructed an efficient 3D-CNN model to achieve accurate classification of VaMCI subtypes. To our knowledge, no similar studies have been reported.

## Materials and Methods

### Participants

A total of 242 subjects with SIVD were recruited from patients admitted to the Neurology Department of Renji Hospital from July 2012 to January 2018. SIVD is defined as subcortical WMH on T2-weighted images with at least one lacunar infarct, in accordance with the criteria suggested by Galluzzi (Samantha et al., 2005). All participants received baseline evaluation, including complete collection of sociodemographic and clinical (cognitive, behavioral, neurological, functional, and physical) data. Patient histories were collected from knowledgeable informants, usually from their spouses. All patients underwent laboratory examinations and conventional MRI for routine investigation (Yao et al., 2019).

The exclusion criteria (Yao et al., 2019) were cerebral hemorrhages, cortical and/or corticosubcortical non-lacunar territorial infarcts and watershed infarcts, specific causes of white matter lesions (e.g., multiple sclerosis, sarcoidosis, and brain irradiation), neurodegenerative disease (including AD and Parkinson’s disease), and signs of normal pressure hydrocephalus or alcoholic encephalopathy. Patients with low education level (<6 years), severe depression [Hamilton Depression Rating Scale (HDRS) ≥ 18], other psychiatric comorbidities or severe cognitive impairment (inability to perform neuropsychological tests), severe claustrophobia, and contraindications to MRI (e.g., pacemaker and metallic foreign bodies) were also excluded. All the participants had lacunar infarcts, small white matter hyperintensities, and slight atrophy.

Finally, all SIVD patients recruited were subdivided based on cognitive status into subcortical vascular disease with no cognitive impairment (NCI) group (*n* = 94) and VaMCI group (*n* = 148). All the participants were right-handed.

The current study was approved by the Research Ethics Committee of Renji Hospital, School of Medicine, Shanghai Jiao Tong University, China. Written informed consent was obtained from each patient.

### Neuropsychological Assessment

Neuropsychological assessments (Yao et al., 2019) were performed within 2 weeks of the MRI. All subjects did not suffer a new clinical stroke or TIA between the MRI and assessment. A comprehensive battery of neuropsychological tests was designed based on a review of relevant published reports. These tests are as follows: Trail-Making Tests A and B, Stroop color–word test, verbal fluency (category) test, auditory verbal learning test (short and long delayed free recall), Rey–Osterrieth Complex Figure Test (delayed recall), Boston Naming Test (30 words), Rey–Osterrieth Complex Figure Test (copy), Lawton and Brody’s Activities of Daily Living (ADL) Scale Test, Barthel index (BI), HDRS, and the Neuropsychiatric Inventory.

To assess the cognitive status of subjects, the scores for each measure of normal-aged patients in Shanghai, China, were used as the normal baseline (norms) (Yao et al., 2019). Cognitive dysfunction was defined as −1.5 SD in at least one neuropsychological test. According to the AHA Statement on Vascular Contributions to Cognitive Impairment and Dementia (Philip et al., 2011), VaD diagnosis was based on a decline in cognitive function from a prior baseline and a deficit in performance in ≥2 cognitive domains that were of sufficient severity to affect the subject’s activities of daily living, which were independent of the motor/sensory sequelae of the vascular event. VaMCI diagnosis was based on the following criteria: (1) ADL could be normal or mildly impaired, (2) does not meet criteria for dementia, and (3) mild quantifiable cognitive impairment within one or more domains (i.e., attention, executive function, memory, language, and visuospatial function). Functional ability was assessed using BI and Lawton and Brody’s ADL scales. However, because most patients with cognitive impairment due to cerebrovascular disease have some degree of disability, the study carefully excluded those with disability due to cognitive damage and motor sequelae using cognitive impairment history and clinical judgment. The definition of subtypes of MCI according to criteria proposed by a multidisciplinary and international experts group includes a-MCI and na-MCI (Winblad et al., 2004). NCI was defined as subcortical vascular disease with NCI, which means their scores in all neuropsychological tests were within the normal range (<-1.5 SD).

### MRI Protocol

MRI was performed with the SignaHDxt 3T MRI scanner (GE Healthcare, United States). An eight-channel standard head coil with foam padding was used to restrict head motion. Besides conventional brain MRI plain scanning, T2-weighted FLAIR sequences with high resolution were acquired as follows: TE = 150 ms, TR = 9,075 ms, TI = 2,250 ms, field of view (FOV) = 256 × 256 mm^2^, matrix = 128 × 128, slice thickness = 2 mm, number of slices = 66.

### MRI Data Preprocessing Pipeline

**FIGURE 1:**
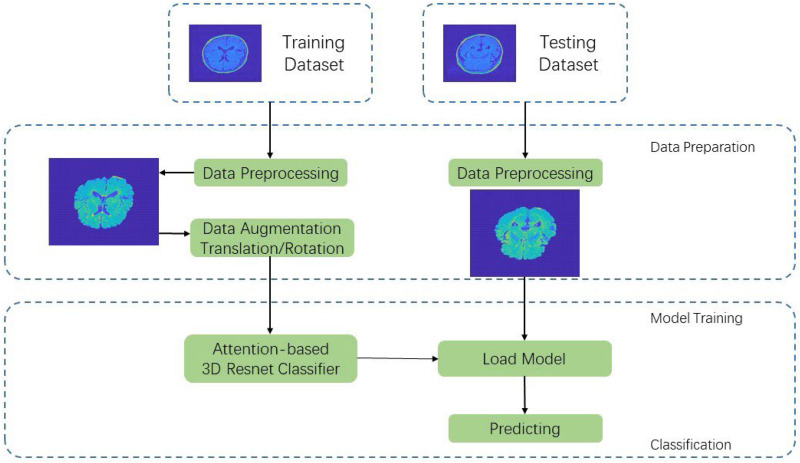
MRI data processing pipeline.

In this section, we propose an end-to-end data pipeline for MR image data processing. The data pipeline contains data preprocessing and model training. Our raw data are T2-weighted FLAIR MR image collected from 242 patients including 78 a-MCI, 70 na-MCI, and 94 NCI. We split the total dataset to three parts including a training set, a validation set, and a testing set with percentage of 60, 20, and 20%, respectively. Figure 1 shows our proposed MRI data processing pipeline. First, we process the raw data using our data preprocessing method and get trainable data as the input of CNN. Then we feed these processed data into our proposed 3D deep residual network to extract higher-level features and carry out the classification procedure. In the following two sections, we will introduce the pipeline in detail. processing pipeline.

### Data Preprocessing

#### Space Conversion

The MRI data are acquired by tomography. It always takes a long time to complete the acquisition of MR images from a patient, and the patient will inevitably move during such a long acquisition procedure. These collected raw tomographic MRI data may not be mapped one by one when aligned and cannot be connected between different slices for effective analysis. Thus, we first process the MRI data into the same data coordination to map different slice layers into standard space. In this paper, we use SPM software and MRIcro software in Matlab toolkit to process these raw MR image data. The specific steps include format conversion, slice timing, head movement realignment, image matching, brain segmentation, spatial standardization, and so forth.

#### Brain Separation Using FSL-BET

**FIGURE 2:**
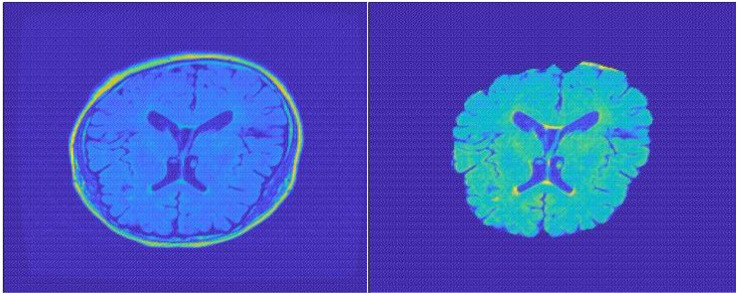
Top view of MR image: the **left** one is before separation; the **right** one after separation.

Traditional sMRI data contain the total brain scanning data including the skull and other non-brain parts, which is meaningless for convolutional networks to extract features. In this case, the skull and non-brain parts act as random noise, and we need to separate them from brain data. In the specific preprocessing process, we used FSL-BET tool to extract the brain structure. We set the fractional intensity threshold to 0.3 and the vertical gradient in fractional intensity threshold to 0.2. The skull separation processing result diagram is shown in Figure 2.

#### Brain Region of Interest Segmentation

We transform the DICOM FLAIR image into mat format in MATLAB with the shape of *l* × *w* × *d* × *c* equaling to 256 × 256 × 66 × 1, where *l*, *w*, *d*, and *c* represent the length, width, depth, and color channels of the image, respectively. Considering that there are still lots of meaningless zeros surrounding the brain region, we define the nonzero brain region as our region of interest (ROI) and use contour finding algorithm to find the maximum ROI part in all slices of samples. We then cut the brain ROI into the size of 159 × 141 × 66. By cutting the ROI, we can focus more on the useful brain region. We can also effectively reduce the number of convolution network parameters, which can speed up the training process as well as reduce the risk of overfitting.

#### Image Smoothing

Noise cannot be completely avoided under any circumstances, and it is similar for medical images. The main noise sources of MR images are thermal/electrical noise and random noise. The most common preprocessing method is to filter the image. In this paper, we use smoothing method in SPM software. We use Gaussian filtered convolution kernel function to convolve the spatial domain of the MR image, so as to remove the high-frequency noise part of the image, leaving the corresponding low-frequency blood oxygen level and other signals in the MR image. Through image smoothing, differential errors in the signal caused by the image capacity and structure of different subjects are eliminated.

#### Data Augmentation

Because the collection of MR images is cumbersome and involves medical ethics issues, the total number of samples in our experiment is 242 and need to be separated into train, validation, and test datasets during model training. The features learned by the model may not have extensiveness and may have serious overfitting problem. In order to solve such problems, this paper refers to the method of data augmentation, which is commonly used for natural images, and it adopts specific-augmentation method for the T2-weighted FLAIR MRI data in this paper. In the data preprocessing process of DL, traditional data augmentation methods mainly aim at the samples of two-dimensional (2D) natural images may have some jitter, noise, and other deviations during the acquisition process. In order to standardize the image, they perform geometric transformation such as translation, flip, rotation, and other augmented transformation. As mentioned above, patients’ head may have slightly shift or rotation in the data acquisition process. Thus, in our experiment, image panning and slight rotation augmentation method are used to augment samples in the training set.

### Convolutional Neural Networks

Medical images are different from traditional natural images in terms of data dimensions and data representation. With the continuous improvement of medical image collection methods and data storage capabilities, the complexity of medical images at the professional level is also increasing. Previously, medical images could only be used as an auxiliary tool for the subjective decision of doctors. Under current situation of increasing density of medical image data, doctors’ experience and ability to judge medical images are difficult to keep up with the pace of image development. However, diagnosis is still based on a traditional knowledge system nowadays.

These advances in medical image data collection have not been applied to clinical diagnosis well, and there is redundancy in medical resources. Thus, it is in great demand to develop new automated clinical diagnosis methods. Previously, a solution to this phenomenon was to use ML to perform prediction, segmentation, diagnosis, and so forth, to realize automated diagnosis process. However, the learning capabilities and models of traditional ML methods are often insufficient to handle such a large number of medical images and high-dimensional data. With the improvement of DL (Lecun et al., 2015) and CNN (Lawrence et al., 1997) and the continuous innovation of computer computing capabilities, a combination of high-performance computers and DL methods can be used to learn and process large-scale medical image data extracted from medical image data and inherent higher-order features of the images.

#### Network Structure

In natural image processing, CNN generally use 2D kernels to implement feature extraction because natural images are mostly 2D. However, MR images are continuous between different slices from the top to bottom. Given that we do not know the exact lesion area of SVCI disease, we use combination of 3 × 3 × 3 and 7 × 7 × 7 three-dimensional convolutional kernels instead of using traditional 2D convolutional kernels to extract 3D features.

Our network uses residual neural network (ResNet)-18 (He et al., 2016) as backbone, which has the best classification effect in 2D natural images and change the structure of the convolution kernel in the model into 3D convolutional kernels so that it can be used for the classification of 3D MR images.

Considering the high density of MRI data in this experiment, our network has a larger number of parameters and a smaller sample size to train this model, which makes it difficult for convergence during the training process. We are inspired by the attention mechanism (Vaswani et al., 2017; Jin et al., 2019) and propose an end-to-end attention-based 3D ResNet model for classification of different subtypes of SVCI on the basis of T2-weighted FLAIR MR images.

Attention model in DL simulates the human brain. When a person is observing a picture, although his or her receptive field can see the entire area of the image, his or her attention to the entire image is not balanced. There is a certain weight to distinguish different regions in human vision, and the effective area that the eyes focus on is actually a very small part. In our experiment, high-density MR image will produce more parameters in neural network. If a model wants to memorize more information of the input image, it has to increase the complexity of the network, which will produce more parameters. This will be a huge burden to our compute capability. Thus, in this paper, we import attention module into our network to focus more on the important region to classify different subtypes of SVCI. In this paper, we use a 3 × 3 × 3 convolution filters activated by ReLU as a subway after convolution feature maps F_*i,c*_ to produce our attention mask A_*i*_. We then multiply attention mask A_*i*_ to previous feature maps F_*i,c*_, so that we can get the weighted attention map M_*ic*_ by the following equation:

$$M_{i\,c}=A_{i}*F_{i\,c}$$

**FIGURE 3:**
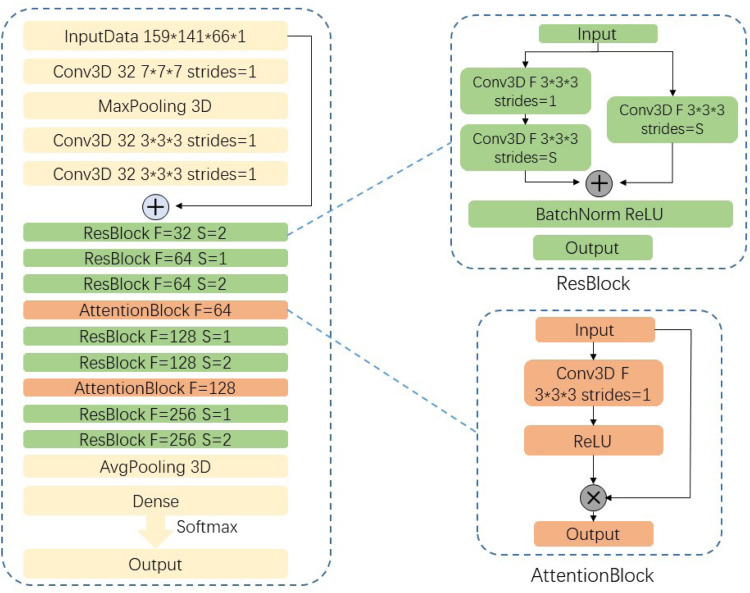
Network structure.

The attention mask A_*i*_ can be trained and optimized through model training to focus more on the significant parts. Our proposed network structure is shown in Figure 3. The network is composed of convolutional layers, ResNet blocks, attention blocks, and output classifier. For example, the Conv3D thirty-two 3 × 3 × 3 strides = 1 layer means 32 convolution filters with the size of 3 × 3 × 3 and strides equal to 1. Different from 2D convolution filters, these filters can receive data from three adjacent slices and can extract features between slices. We fed our preprocessed data with resolution of 159 × 141 × 66 × 1 into the network and go through eight residual blocks. As the layers go deeper, the numbers of filters will increase from 32 to 256, and the features extracted will be more abstract and complex. Correspondingly, the last layers in ResBlock have parameter *S* set to 2, which means that we set the strides to 2 × 2 × 2 and downsample the feature maps size by two times. Then the last output feature maps will be average-pooled and fed into the classifier.

#### Experiment Settings

We implement the experiment on two NVIDIA GTX 1080 Ti GPUs. We applied the *k*-fold cross-validation method in training. The total dataset are divided into five equal shares; and for each training process, we use four shares as training and validation sets and one share as the testing set. The final test accuracy and other metrics are calculated by the average of five experiments. The experiment is based on Keras using TensorFlow as backend. Limited by the computation ability, our batch size is set to 4. In our network, preprocessed data with the shape of 159 × 141 × 66 × 1 are fed and are filtered by gradually increasing filters to extract high-level features. The features are finally fed into a fully connected (FC) layer activated by softmax to get the final classification output. We use cross-entropy loss function and adaptive gradient algorithm (Adagrad) optimizer to help our model minimize the loss function. Cross-entropy loss function is shown as follows:

$$L_{\log}\,\left(Y,P\right)=-\log\mathrm{Pr}\left(Y|P\right)$$

$$=-\frac{1}{N}\,\sum_{i=1}^{N-1}\sum_{k=0}^{K-1}\left(y_{i\,k}\right)\,\log\left(p_{i\,k}\right)$$

where multivariate classification **k** is the total number of categories, y_*ik*_ equals to 1 only if the label of the *i*-th sample is in category **k**, the true category label of **N** samples is an *N* × *k* matrix **Y**, and the probability of each sample in **N** samples predicted by the classifier is an *N* × *k* matrix **P**.

The updated formula of Adagrad is shown below:

$$\theta_{t+1,i}=\theta_{t,i}-\frac{\eta }{\sqrt{G_{t,i\,i}+\varepsilon }}$$

where *g* is the gratitude at time θ_*i*_; in our experiment, we set η as 0.01. Adagrad can do larger updates for low-frequency parameters and smaller updates for high-frequency and can solve the problem that different parameters cannot be updated to different scales according to the importance of the parameters.

## Results

**FIGURE 4:**
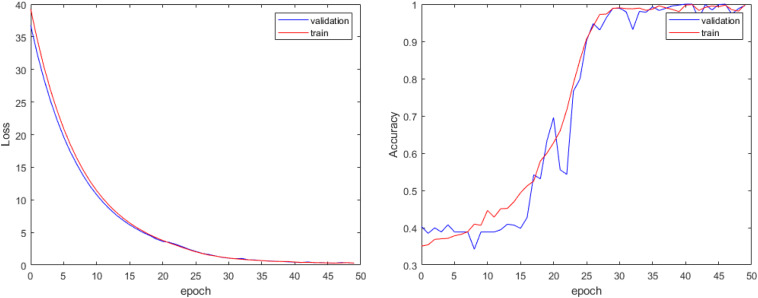
Loss curve and accuracy curve of our model.

In our experiment, we train the proposed attention-based 3D ResNet for 50 epochs. Because there are no relative pretrained models in our classification of different subtypes of SVCI with FLAIR MR image, we train our model with random initialization. With proper hyper-parameter tuning, we approach the best performance on the training set and validation set as shown in Figure 4.

Because there are no such methods for the classification of different subtypes of SVCI, our proposed model has significant clinical value. The accuracy of our proposed model on the testing set reaches 93.8% with robustness. Thus, our proposed method can effectively assist doctors in early detailed classification diagnosis, so as to carry out targeted treatment in time. In addition to test accuracy, we also consider three other indexes for comparison. We introduce recall (*R*), precision (*P*), and F1 score. Precision is the ratio of true positive samples to predicted positive samples, and recall is ratio of true positive samples to actual positive samples. These two indexes can be combined to F1 score as a thorough evaluation of the classification. The formulas of these three indexes are as shown below:

$$R=\frac{T\,P}{T\,P+F\,P}$$

$$P=\frac{T\,P}{T\,P+F\,N}$$

$$\frac{2}{F\,1}=\frac{1}{R}+\frac{1}{P}$$

where TP, FP, FN, and TN represent positive samples classified to positive, negative samples classified to positive, positive samples classified to negative, and negative samples classified to negative, respectively. Because our experiment is a three-category classification problem, we consider one category as positive samples and the other two as negative samples each time.

The final performance of the model is shown in Table 1:

**TABLE 1:** Three other index performance of proposed method under three subtypes of subcortical vascular cognitive impairment.

Subtypes	Recall/%	Precision/%	F1 score/%
A-MCI	93.2	91.9	92.6
NA-MCI	94.3	94.3	94.3
NCI	93.8	94.7	94.2

## Discussion

Using 3D convolutional kernels, we successfully trained an efficient CNN model that could accurately classify different subtypes of VaMCI (a-MCI and na-MCI) as well as NCI by extracting 3D features from raw T2-weighted FLAIR brain scans. The accuracy of the training set and the testing set reached 98.9 and 97.3% after 50 epochs, respectively. It furthered our previous work of classifying different cognitive performances in SIVD, which is also based on single FLAIR sequence (Yao et al., 2019). These two studies together proved that the method of 3D CNN combined with high-resolution sMRI was worth applying in clinical evaluation of small vessel disease in the elderly. FLAIR sequence was used in our study because it could maximally reflect the imaging features of SVCI such as lacunar infarct and WMH, and the result finally verified the validity of the sequence.

Nowadays, neuroimaging examination has become an indispensable part of clinical evaluation in SVCI, especially MRI with a variety of advanced sequences such as DTI, susceptibility-weighted imaging (SWI), functional MRI, and perfusion-weighted imaging. However, as a result of the imbalance of patients’ benefits from the expensive and time-consuming MRI examination, there is still a lack of methods worthy of promotion for the accurate diagnosis and evaluation of patients. DL offered us an opportunity to obtain high clinical diagnostic accuracy with even one single sequence, for it can take full advantage of spatial contextual information in MRI volumes to extract more representative high-level feathers. It could greatly shorten the MRI examination time, reduce the patient’s stress caused by the long-time examination, avoid the use of a large number of expensive advanced MRI sequences, and simplify the complex and time-consuming postprocessing. It is important to note that in order to get high-quality image information, we collected high-resolution FLAIR images, which cost 6 min 30 s. Whether thick-layer images as a clinical diagnosis most often used could achieve similar accuracy needs further research. Considering that high-resolution MR imaging data consist of numerous slices that have a continuous spatial positional relationship, we applied a 3D-based CNN model rather than a 2D-based network, which has been proved to be more efficient in our previous study (Yao et al., 2019). Finally, we got a high accuracy of subclassifying VaMCI into a-MCI and na-MCI.

The subclassification of MCI has clinical significance, because different MCI subtypes may subtend different etiologies. a-MCI may indicate a degenerative etiology and has higher dementia risks than has na-MCI, whereas na-MCI may indicate a vascular etiology that needs more treatment to improve vascular function and cerebral perfusion (Liu et al., 2018, 2019; Yang et al., 2020b). On the basis of single high-resolution FLAIR images, we proved that 3D-CNN can classify not only different cognitive impairment stages in SIVD but also subtypes in MCI stage. This method can greatly improve the efficiency and accuracy of clinical diagnosis of SVCI and is beneficial to clinical targeted treatment at the early stage of cognitive impairment.

Although we have achieved an appealing performance with a high accuracy in this study, there are still several limitations. First, this is a retrospective study with a relatively small sample size. Large-scale multicenter and perspective studies are needed to fully assess the generalization ability of the model. Second, more detailed clinical groups such as single domain and multidomain cognitive groups with or without amnesia based on sufficient sample size can further test this 3D-CNN model and enrich its clinical application. Third, the clinical or pathological interpretation of the association between the high-level features and the cognitive performances remain challenging. Further studies are needed to establish a rationale to explain the correlation between deep imaging features and cognitive performances, which might hint at the underlying pathological mechanisms of SVCI.

## Conclusion

In this paper, we proposed an end-to-end attention-based 3D ResNet model for classification of different subtypes of SVCI with T2-weighted FLAIR MR images. End to end means doctors do not need to perform complicated data preprocessing; they can simply input the single MRI scanning image of patients to the model and get the output of SVCI classification. Then they can further get the diagnostic decision results according to the auxiliary diagnosis results of our proposed methods. Our proposed method provides a convenient and effective way to assist doctors in the diagnosis and early treatment.

## Data Availability Statement

The datasets generated for this study are available on request to the corresponding authors.

## Ethics Statement

The studies involving human participants were reviewed and approved by the Research Ethics Committee of Renji Hospital, School of Medicine, Shanghai Jiao Tong University, China. The patients/participants provided their written informed consent to participate in this study.

## Author Contributions

YZ and GZ designed and instruct the experiments. QC wrote the code for the experiments. QC and YW carried out the experiments and wrote the manuscript. YW, YQ, and XW collected and analyzed the experiment data. All authors listed have made a substantial, direct and intellectual contribution to the work, and approved it for publication.

## Conflict of Interest

The authors declare that the research was conducted in the absence of any commercial or financial relationships that could be construed as a potential conflict of interest.
